# Howard P. Roffwarg: sleep pioneer, legend, and ontogenetic hypothesis author

**DOI:** 10.1093/sleepadvances/zpad004

**Published:** 2023-01-23

**Authors:** James P Shaffery, Gerald A Marks

**Affiliations:** Department of Psychiatry and Human Behavior, University of Mississippi, Jackson, MS 39216-4505, USA; Department of Psychiatry, University of Texas Southwestern, Dallas, TX 75390, USA

**Keywords:** rapid eye-movement sleep, ontogenetic hypothesis, brain development

## Abstract

Narrated in this article are accounts of the many contributions Howard P. Roffwarg, MD, made to the field of sleep research and sleep medicine across his entire professional career as a student, a mentor, a leader in the Sleep Research Society, a sleep medicine clinician, and a scientist who performed experimental investigations in humans and animals. Dr Roffwarg was the originator of what is known as the “Ontogenetic Hypothesis” of sleep. His research over many years on physiology has contributed greatly to much of the experimental support substantiating a role for rapid eye-movement sleep (REMS) in the early development of the brain. Though much is still unknown, the Ontogenetic Hypothesis, still to this day, inspires many neuroscientists in their investigations. These studies have demonstrated roles for both REMS and NREMS in development as well as on brain function throughout his life span. Dr Howard P. Roffwarg, is one of the legends in the field of sleep research.

**Statement of Significance**

This article documents the many contributions Howard P. Roffwarg, MD, has made to the field of sleep research and sleep medicine. It spans his entire professional career highlighting his multiple roles as student and mentor, in leadership in the Sleep Research Society, his work in sleep medicine, and in experimental investigations in humans and animals. Dr Roffwarg was the originator of what has come to be known as the “Ontogenetic Hypothesis.” His research over many years on physiology has contributed greatly to much of the experimental support substantiating a role for rapid eye-movement sleep in the early development of the brain. Though much is still unknown, the ontogenetic hypothesis has, and is still to this day, inspired many neuroscientists in their investigations.

We describe here the many contributions Howard P. Roffwarg has made to the field of sleep research and sleep medicine throughout his career. Dr Howard P. Roffwarg, is one of the legends in the field of sleep research and his enduring pursuit of the “Ontogenic Hypothesis” of sleep. With the discovery of the dual, alternating, and distinct states of sleep, rapid eye movement sleep (REMS) and slow wave sleep (SWS or non REMS, NREMS) [1], it was no longer tenable to hold the age-old view that the function of sleep is solely for rest and recovery from the physiological demands of wakefulness. Particularly challenging to this view is the finding that REMS is not a quiescent state but rather one in which the brain is highly active, often rivaling that found in wake [2, 3]. NREMS also expresses unique activities that differ from both wake and REMS. To this day, scientific inquiries into the mechanisms and functions of sleep-specific central nervous system activities are the major questions of basic sleep research.

In the mid 1960s, Howard P. Roffwarg, a young psychiatry resident working in the William Dement’s laboratory, was polygraphically recording human infants to determine the natural development of the sleep/wake cycle. He found that human newborns, known to sleep most of the day, have a mean time of 16 h of sleep and that 50 percent of this time is spent in REMS. In comparison, 19–30-year-olds have a mean daily sleep time of 8 h per day and 22 percent in REMS. The reduction in time between these ages is approximately 80 percent for REMS and 25 percent for NREMS. This finding was an unexpected result at the time (see Figure 1). The high volume of REMS in the young compared to adults has been since demonstrated in numerous mammalian and avian species and appears to be a generalized phenomenon [4, 5].

**Figure 1.**
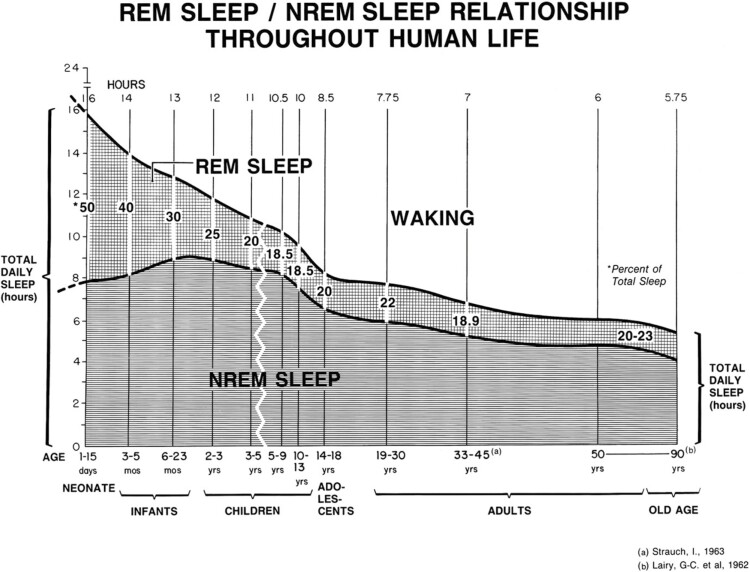
Graph showing changes (with age) in total amounts of daily sleep, daily REM and NREM sleep, and in percentage of REM sleep. This figure appeared in the Science paper authored by Roffwarg, Muzio, and Dement, 1966, reporting the ontogeny of Human sleep across six life spans (with permission from the publisher).

The strong negative correlation between the degree of brain maturation and the time spent in REMS prompted Howard to posit a causative relationship between the high amounts of endogenously driven REMS-specific neural activity and the physiological processes of brain maturation. He reasoned that in early life, the dependence of exogenously driven neural activity in both sensory and motor systems required for normal development, occurs only during an attenuated time period, wake, and so could be greatly augmented by endogenously driven neural activities that occur specifically during the respective two stages of sleep. He further conjectured that sleep, though appearing in lower amounts in adults, may perform several important functions through the life span by the states’ control of neural activity.

These findings and compelling supportive arguments were presented in the journal Science in 1966, in a paper titled “Ontogenetic Development of the Human Sleep-Dream Cycle,” authored by Roffwarg, Muzio, and Dement [6]. The paper has been cited in over 1400 scientific articles and is known in the field of sleep research as the “Ontogenetic Hypothesis.” It is one of the most enduring concepts of a possible function for either of the two sleep states (see also [7]).

Howard P. Roffwarg earned an AB in 1954 from Columbia University New York and his MD in 1958 from the College of Physicians and Surgeons Columbia University. This was followed by a medical internship at Stanford University Hospital (California) from 1958 to 1959, and, starting in 1959, a Residency in Psychiatry at New York State Psychiatric Institute, Columbia University. Howard’s educational experience prepared him for a trajectory directed towards the practice of clinical medicine in psychiatry, in which he found great satisfaction and pursued it throughout his entire professional career.

One day early in his residency, he passed a bulletin board announcing a lecture to be given at the New York Academy by Dr William Dement entitled “Dream Deprivation.” What he heard was a lecture on the relatively new finding of REMS, its deprivation, and rebound. Howard was so fascinated by the talk that following the lecture he made his way to the front and, with trepidation, requested to work in Dr Dement’s laboratory during a research rotation. At the time, Dement was building a new sleep laboratory and asked Howard to get back in touch in a few months. After several telephone conversations, Howard’s eagerness was not diminished, and Dement finally offered Howard a research position. This resulted in a two-year research associate position in the Mount Sinai Hospital Laboratory of Drs William Dement and Charles Fisher. This experience led to a friendship with Bill Dement that he cherished throughout his life, and a career trajectory that now shared sleep research as a major effort alongside his commitment to his clinical practice in Psychiatry. At Dement’s invitation, Howard attended the first Sleep Research Society (SRS) meeting in Chicago (1961) as a trainee and was, thus, one of the founding members of what is now the SRS. Howard was awarded an NIMH career development award while in Dement’s laboratory that funded polygraphic recording in human infants generating data for the basis of the ontogenetic hypothesis of REMS function.

Following the research associate position in Dement’s laboratory, Howard, as an independent investigator, was appointed director of the Sleep EEG Laboratory at the New York State Psychiatric Institute, and later as director of the Sleep Research Laboratory, Department of Psychiatry Montefiore Hospital (1962–1977). At Montefiore, Howard developed both human and animal research laboratories. He collaborated with the Steroid Institute and Elliot Weitzman resulting in many studies on human hormone secretion, circadian rhythms, and sleep. He received the Anna Monika Foundation Award (2nd prize) in Neuroendocrine Studies in Depression in 1975. From 1967 to 1971, Howard served on the Brain Information Service, Committee of Terminology, Technology, and Scoring in Sleep Research which resulted in the publication of the first standardized scoring systems for human sleep [8]. From 1968 to 1971, he was a member of the Executive Committee, Association for the Physiological Study of Sleep (now the SRS).

During his time at Montefiore, Howard sought to further expand the scope of his sleep neurophysiological investigations. He contacted Michel Jouvet in Lyon, only to find that Jouvet traveled during summer, the only time Howard could find to go. Jouvet suggested Francois Michel at the Faculte des Sciences, Universite Lyon. Michel was a colleague of Jouvet’s and adept in using the split-brain preparation in cats. Howard spent two summers with Michel (1965, 1966) as a visiting Research Associate, learning the techniques and studying the effects of the surgical lesions on sleep–waking cycles in cats. Conversations and friendships were valuable assets of this experience, including lengthy conversations with Danielle Jouvet-Mounier, who occupied the laboratory next door to Michel’s. She was investigating sleep ontogenesis in a variety of mammalian species, and her work confirmed Howard’s finding in humans of high amounts of REM sleep in neonates. Another colleague was Joelle Adrien, then from Lyon, who came to do a fellowship in the laboratory at Montefiore investigating the ontogenesis of sleep-related unit activity and pontogeniculoccipital (PGO) waves in the lateral geniculate nucleus (LGN) of kittens. Other investigations in Howard’s animal and human laboratories at Montefiore included interactions between sleep and visual perception, dream imagery, REM sleep PGO activity in the auditory system in both humans and cats, and REM sleep PGO activity in rats. Collaborators included Joelle Adrien, John Herman, Jorge Farber, a postdoctoral fellow, and Gerald Marks, at the time a graduate student at The City University of New York. From 1972 to 1975, Howard served as Associate Editor and then Editor-In-Chief of Sleep Review, Brain Information Service.

In 1977, Howard’s sleep laboratories underwent major transformations. Kenneth Altshuler (of Columbia University) had recently taken the position of Chairman of Psychiatry at the University of Texas Health Science Center in Dallas, TXs (to become UT Southwestern Medical Center, UTSW). Dr Altshuler offered Howard a position—one he, apparently, could not refuse. Howard received appointments as Director, Sleep Study Unit and Director of Research, Department of Psychiatry. It was one of those cyclic Texas boom-times that included new buildings, new, fully equipped laboratories, and many slots for personnel. Joelle had left Montefiore earlier to take a position in Paris, but John, Jorge, and Gerry came along for the exciting ride. Howard and his group were not the only “new kids on the block”; a large number of new faculty recruits from institutes around the country swelled the ranks of the department of Psychiatry. New opportunities abounded for collaborations.

One long-lasting and prolific collaboration at UT Southwestern was with AJ Rush and DE Giles, accomplished investigators in the field of affective disorders. Howie also formed another significant collaboration there with Graham Emslie, studying sleep in childhood and adolescent depression. Utilizing polygraphic recording, many important findings were uncovered investigating the relationship between major depressive disorders and REMS latency. John Herman continued his collaboration with Howard on human-altered visual experience and REMS, including a study on the relationship between eye movements and dream imagery. John left the sleep study unit around 1983 for a codirector position in sleep medicine at the Dallas Children’s Hospital.

In Howard’s animal laboratory, Gerry Marks and Jorge Farber pursued studies of sleep-related neuronal activity in several thalamic nuclei, with a long-term goal of identifying neural activity interacting with brain development. Their first study demonstrated a temporal relationship between the discharge of LGN units and PGO waves recorded in the hindbrain (70% of units) of rats. Inasmuch as PGO waves cannot be recorded in the LGN of rats, this neural activity represents another phasic event of REM sleep, analogous to PGO-waves seen in cats [9, 10].

During this time, Howard, Gerry, and Jorge had weekly discussions on how to experimentally approach testing the ontogenetic hypothesis. Many laboratories had, and were, pursuing Howard’s ontogenetic hypothesis. Many of these studies, though supportive, relied on a series of drug injections (mostly antidepressants) that, while suppressing REM sleep, would also be expected to have nonspecific consequences when administered during a period of widespread brain regions undergoing maturation. This turned out to be accurate. Recent data have shown that treatment in early life with selective serotonin reuptake inhibitors (SSRIs), leads to permanent alterations in dorsal raphe neurons [11, 12]. They decided to use an instrumental-type of selective REMS deprivation (REMSD), one that would accomplish a specific suppression of both tonic and phasic neural activity expressed during REMS by not allowing the REMS state to occur.

The next decision was to choose the dependent variable. From the pioneering work of Hubel and Wiesel [13, 14], it had been shown that normal development of the visual system during a critical period (CP) of visual development in kitten maturation (postnatal day P14–P90 [15]), is dependent on visual experience. Hubel and Wiesel, along with others by that time, had documented how manipulating visual experience, such as patching one eye to cause monocular deprivation (MD), resulted in morphological alterations to the size of neurons in the LGN. The LGN is the thalamic relay of inputs from the retina on to visual cortex. Neurons located in the LGN binocular segments are sensitive to cell-size changes after MD. Binocular segment cells receive retinal inputs from the visual field shared by both eyes and constitute the two dorsal layers of cells in LGN. The most dorsal, lamina A, only receives input from the contralateral eye and the layer just below, lamina A1, only from the ipsilateral eye. MD during the CP results in significant reductions in cell-sizes in the lamina receiving input from the occluded eye, while the cells receiving input from the seeing eye tend to be larger. Inasmuch as the cell-size alterations resulting from MD during the CP are a consequence of a physiological developmental process, demonstrating interactions with alterations in REMS activity, would reveal REMS’s involvement with this same developmental process.

The remaining piece of the puzzle was to ensure that each subject had the same visual experience in that visual experience itself affects development. This was accomplished by altering the light–dark cycle to 1.5 h - on, followed by 4.5 h - off. Kittens would be active and playing in the light and subjected to the REMS deprivation (REMSD) or control conditions in absolute darkness. This procedure equalized waking light experience in all subjects to 6 h per day, the published average for unmanipulated kittens of this age. The basic concepts of the experimental protocols were finalized utilizing development of the visual system following MD, with alterations to cell-size in the LGN binocular segment as the dependent measure and REMSD in kittens during the CP the independent variable.

Just as the ontogenetic studies were about to begin, Jorge retired from science for personal reasons. With only Gerry and Howard on board, there was a critical need to increase personnel. Agreements were made with two UT faculty members, Sam Speciale (biochemist) and Greg Mihailoff (anatomist). These collaborations gave the sleep team the expertise in microscopy needed to perform the required cell-sizing. The still-needed increase in personnel was met by Arie Oksenberg. Arie was working with cats as a graduate student with Rene Drucker-Colin in Mexico City. Looking ahead for a postdoctoral position, Arie was very interested in the relationship between sleep and brain development and thought that working in Howard’s lab in Dallas would be a good fit. He also was impressed when Rene described Howie as a “really nice person.” Arie’s first impression of Howard didn’t quite play out that way. Arie “cold-called” Howard from the Dallas airport, introduced himself, talked about his research interests, and asked if he could meet with Howie that same day. Howard was taken aback by the cold call and explained to Arie that “this is not the way you meet people!”. Howard did arrange for Arie to meet the sleep team members that day and made time to see him at the end of the day, exhibiting his usual demeanor as a really nice person. Arie moved to Dallas to assume a postdoctoral position in the laboratory. This was the team that started Howard’s second chapter on his investigations of the ontogenetic hypothesis.

In these initial experiments, kittens were subjected to instrumental REMSD, by the multiple-small-platform-over-water method. Control kittens on large platforms expressed normal amounts of REMS [16]. These procedures were during the last 7 days of a 2-week period of MD corresponding to the peak of the CP (P35 to P49). Data, first published as abstracts in Sleep Research [17, 18], indicated that REMSD, combined with MD, led to more extreme changes in LGN relay cell-size in the binocular segments compared to control kittens. Data from these control (MD-only) animals, replicated published results from earlier vision research, LGN cells either increased or decreased in size, according to whether the cell was receiving input from the nonpatched or patched eye. In the MD animals that also experienced REMSD, LGN cell size changes were amplified compared to the MD-only control animals. Removing LGN activation from both visual and REMS-related activity amplified the effects seen in MD-only animals. This amplification of LGN cell-size differences with REMSD strongly suggested that REMS, when normally present plays a part in normal processes of maturation in the visual system. This added strong support to the basic tenets of Howard’s ontogenetic hypothesis.

Along with Gerry and Sam, Arie contributed a great deal to these initial studies in kittens, having conducted the kitten LGN cell-sizing studies that would underlie both their forthcoming (at the time) binocular- and monocular-segment papers [19, 20]. Shortly after this early work, however, Arie decided that his future was in studying sleep disorders in humans, and in 1987 he returned home to Israel. To continue the Ontogeny studies, a new postdoctoral fellow was needed. Howard attended the 1988 SLEEP meeting, in part, with the goal of identifying a new postdoctoral fellow to carry on with the ontogeny studies. It was at this meeting that Howard first met Jim Shaffery, after having met with Roseanne Armitage. Howard and Roseanne were finalizing details of her taking an Assistant Professor position at Dallas to lend her expertise with new analysis strategies for analyzing sleep EEGs in Howard’s human sleep laboratory. Roseanne said she might know of a postdoctoral candidate and arranged for Howard to meet Jim at lunch the next day. Jim had just completed a postdoctoral fellowship position with a group studying the function of sleep in free-ranging seagulls. At their meeting, Howard invited Jim to visit the laboratory in Dallas and interview Gerry and Sam. Jim joined the animal sleep laboratory in August 1988, and after learning the techniques of the surgical procedures and cell-sizing in the LGN, he essentially picked up where Arie left off.

With Jim onboard, the animal sleep group expanded the preliminary data Arie helped collect and was ready for final publication [19, 20]. During this period, the animal sleep group also conducted several new experiments to further support their initial findings by addressing a number of questions raised by these data in kittens (described below) and exploring new models testing the ontogenetic hypothesis in young rats.

A subsequent study [19] looked specifically at a different area in the A-lamina of each LGN that receives retinal input exclusively stimulated by the temporal margin of the contralateral visual field that cannot be viewed by the ipsilateral eye. Consequently, the two peripheral field areas do not have anatomical representations in either of the A1-laminae, which exclusively represent the ipsilateral eye. Hence, these portions of the A-laminae are appropriately named the monocular segments. Developmental processes, with respect to different cell-size changes, occurred in binocular and monocular segments. MD produces large cell-size disparities in the binocular segments, while removing all visual stimulation, such as with dark rearing has little effect. Conversely, MD has little effect in the monocular segments, while dark rearing produces significant reduction in cell-size. Study of the monocular segment provides an opportunity to observe effects of REMSD on different developmental processes.

This study compared cell sizes in both LGNs between REMSD+MD kittens and control MD animals. The monocular segment cells associated with the “seeing” eye received equal amounts of waking light experience in both REMSD and control conditions. These data showed that, despite reported ineffectiveness of MD in monocular segment cells, the cells were significantly smaller in the REMSD+MD compared to the MD-only animals. That is, cells in the monocular segment exhibited significant reductions in size after REMD independent of whether they received input from the occluded eye or not, though reductions were significantly greater in cells receiving input from the occluded eye compared to that of the unoccluded eye. The bilateral cell-size reductions in the monocular segments are reminiscent of the effects of dark-rearing. Without the knowledge of the specific mechanisms involved, these results are difficult to fully interpret, but it is apparent that by removing the endogenous activity occurring in REMS, an interaction occurs with developmental processes present in the monocular segment and the binocular segment of the LGN [20]. This further suggests that REMS, when normally present, plays a part in two different, normal processes of maturation in the visual system. And lends further support towards the validation of Howard’s ontogenetic hypothesis.

In another study, utilizing the LGN cell-sizing paradigm, initial findings were challenged with a procedure that suppressed *only* REMS-related ascending phasic activation. The REMS state is comprised of two, semi-independent activities, phasic and tonic [21, 22]. Discrete, bilateral electrolytic lesions at the pontomesencephalic isthmus in very young, normally sighted kittens have been shown to block phasic PGO-wave activity in LGN during REMS and also to significantly slow LGN maturation [23–25]. Specifically, lesions of brainstem, putatively cholinergic ascending pathways suppress PGO-wave activation to LGN and result in reduced overall LGN volume, unit firing rate and cell size, bilaterally, in both LGNs, without altering the normal durations and circadian timing of the REMS state or its tonic activation of thalamic and cortical visual sites [23–25]. The current study extended lesion-induced interruption of REMS phasic activation by coupling it with MD in kittens to test whether doing so would replicate the findings using REMSD. The results showed that the combined effects of the PGO-pathway lesion-*plus*-MD protocol yielded results in binocular-segment cell-size changes equivalent to those seen in their earlier, REMSD study in MD kittens [26]. These results support the hypothesis that CNS activation during REMS plays a role in the development of the brain. It also allows identification of a specific type of REMS activity necessary to the observed developmental effects in LGN attributed to REMSD.

It is apparent that cell-size changes in response to MD in the binocular segment are different from those in the monocular segment. This can be explained by the involvement of the LGN binocular segment in the function of depth perception. Depth perception requires both eyes to focus on an object. When this occurs, the eyes converge eliminating any disparity in the image registered on each retina. This also results in the alignment of the retinal projection across the A and A1 cells of the binocular segment. Information on this alignment and the convergence of the eyes are used by the brain to determine the distance of the object. Cells in the monocular segments of the LGN do not perform this function. These cells only receive retinal input from the contralateral temporal visual field seen only by each eye, respectively. Thus, the innervation is always binocularly disparate.

Their functional role in depth perception makes the binocular segment cells more susceptible to MD during early development. With MD, there can be no alignment across the A and A1 laminae. A hypothesized competitive mechanism has been proposed [27] to account for cells receiving input from the occluded eye to reduce in size, while the size of cells receiving input from the unoccluded eye increase. This mechanism is absent in the monocular segment. The effect of MD in the binocular segment appears to be strong enough to mask the cell-size reductions as observed in the monocular segment. PGO-pathway lesion-*plus*-MD replicates the results of REMSD-*plus*-MD, but PGO-pathway lesion alone results in only cell-size reductions in the binocular segment [20].

Toward the end of this very productive period (ca. 1995), Howard was offered a position in the Department of Psychiatry and Human Behavior at the University of Mississippi Medical Center (UMMC) School of Medicine. Howard accepted a tenured professorship and the Directorships of the Sleep Disorders Clinic, Division of Sleep Medicine, and Sleep Neurophysiology Laboratory. He was joined by Jim, and they were given more than ample space and support for the animal sleep research laboratory to carry on with the now fully funded ontogenetic study. Gerry and Sam chose to stay on at UT Southwestern, but continued to collaborate with Howard and Jim, completing the final analyses for several of the kitten publications, and starting new studies of the effects of REMSD on developmental synaptic plasticity in young rats. The initial journal publications were some of the first results strongly implicating REMS as having a functional role in brain development in support of Howard’s ontogenetic hypothesis. Their work at UMMC also began to identify some of the mechanisms that could account for how REMS-related neuronal activity affects brain maturation.

Effects of REMSD, without using a visual manipulation, were assessed in CP kittens. In the cat visual system, nearly all parvalbumin-immunoreactive neurons are GABAergic interneurons that are involved with synaptic plasticity [28, 29]. In the kitten LGN, these neurons are absent at birth and do not develop until around the time that REMSD is deployed, near the middle of the CP in kittens. These studies showed that REMSD in CP-kittens reduces the number of LGN cells immunoreactive for the calcium-binding protein, parvalbumin, in GABAergic neurons in the LGN compared to normally sleeping CP-kittens [30]. The relevance of this finding is that GABAergic, parvalbumin-containing interneurons modulate some forms of synaptic plasticity [31]. This finding is also consistent with the possibility of REMSD causing a developmental delay in kitten’s visual system similar to that seen after dark-rearing or binocular occlusion (see below).

Taken together, the early kitten work demonstrated REMSD during development abnormally increases several related forms of synaptic plasticity in the visual system [19, 20, 26, 32]. These findings are interpreted to mean that activation of the central visual sensory system during REMS and its exogenous activation by visual experience in the waking animal, both appear to contribute to the configuration of synaptic connectivity in the postnatal CP kitten. Studies described below suggest that the final functional form of the developing visual system as well as the closure of the CP for developmental cortical synaptic plasticity will not proceed to completion in the absence of either of these two activating inputs to the visual system (c.f. [32, 33]).

Developmental plasticity in the visual system has been reported to share mechanisms with several types of experimentally produced models of synaptic plasticity, including long-term potentiation (LTP) and long-term depression [34–39]. LTP is an established model of the activity-dependent synaptic plasticity essential for neocortical development [33, 34, 40–42]. Several different forms of LTP in visual cortex are age-dependent, but others are not. It is significant that the developmental time course of age-dependent forms of LTP in visual cortex is coincident with the CP for visual system maturation [33, 42–45].

Accordingly, one form of developmentally regulated LTP in rats is produced in layers II/III of visual cortex after stimulation of the underlying white matter, the axon fibers from the LGN (LTPWM-III). This normally occurs only during the rat CP (approximately P17 and P30) [33, 44]. After P30, white matter stimulation, alone, seldom evokes LTPWM-III [32, 44, 46]. Several pharmacological and behavioral treatments are able, however, to re-establish or extend this plasticity. For example, developmental LTPWM-III can be obtained in adults when combined with gamma-aminobutyric acid (GABA)-A-receptor antagonists [43, 47] or blockage of nerve growth factor [48]. Another treatment that extends the CP is raising young animals in complete darkness [49–51]. Kirkwood and colleagues showed that dark-rearing, started before the time of eye-opening, maintains the inducibility of LTPWM-III well beyond the usual end of the CP [33]. In effect, dark-rearing maintains the central visual system in an immature state, relative to the time when LTPWM-III can be produced [33]. Inasmuch as both manipulations, dark-rearing and REMSD, postpone visual system maturation, investigations were undertaken by Howard’s sleep research group to determine whether REMSD would have effects like those of dark-rearing on the production of LTPWM-III.

Howard’s group discovered that exposure to REMSD prolongs the inducibility of LTPWM-III in young rats, replicating the results of Kirkwood’s dark-rearing study [33]. The group found that LTPWM-III could still be reliably produced in animals that had been REMSD for 7 to 10 days. Control rats, not deprived of REMS, did *not* produce LTPWM-III during this post-CP period. Pointing to the specificity of the REMSD effect, a nondevelopmentally regulated form of LTP in visual cortex was readily produced in all the rats tested in both groups [32]. These data support the interpretation that REMSD extends the CP for production of LTPWM-III. While questions remain as to whether REMSD and dark-rearing extend the maturational processes underlying the CP via the same or related mechanisms, it appears that REMS is *as necessary* as retinal sensory experience to enable the typical termination of the developmental CP for LTPWM-III. The question remained whether REMSD is capable of reopening CP-plasticity in a fully mature animal. A follow-up study explored whether REMSD in adult rats could reopen synaptic plasticity mechanisms necessary to the induction of LTPWM-III. Using adult rats (>P60 at testing), it was shown that LTPWM-III could not be produced in either control or REMSD rats deprived by the multiple small-platform-over-water method for 7 to 10 days [16, 52]. These two studies showed that REMSD can extend the CP for LTPWM-III, but apparently REMSD does not reopen the developmental synaptic plasticity underlying production of LTPWM [32]. What remained inconclusive is when the potential for LTPWM-III is ultimately extinguished.

Howard’s sleep group next identified a so-called “interim period” that begins shortly after the CP closes, and persisting through adolescence until nearly P60 [53]. They showed that 3 to 10 days of REMSD in “interim-period” rats (P35–P60) allows production of LTPWM-III in almost all animals tested (88% of trials) [53]. Though the conventional termination of the CP for LTPWM-III had passed, a few days of REMSD seemed to able to “rescue” or reestablish the molecular mechanisms necessary to evoke LTPWM-III [53]. In keeping with Howard’s ontogenetic hypothesis, these data strongly suggested that REMS participates in the closure of the CP.

It has been suggested that the closing of the CP for producing LTPWM-III follows upon maturation of a postulated “inhibitory gate,” that is, an inhibitory, presumably GABAergic mechanism in layer IV in visual cortex that either permits or blocks, depending upon relative state of maturity, veridical transmission of WM activity to higher layers of the cortex [36, 54, 55]. Howard’s group wanted to know whether REMS interacts with this “inhibitory gate,” and sought to determine if REMSD affects this mechanism to extend the CP. Considerable evidence links activity-dependent neurotrophic effects to the maturation of cortical GABAergic mechanisms [56]. The sleep group examined this idea in a new set of experiments in young rats near the end of the CP. They tested the hypothesis that REMSD blocks production of brain derived neurotrophic factor (BDNF), and investigated whether replacing BDNF prevents induction of LTPWM-III. This experiment showed in REMSD CP rats, that infusion of BDNF blocked production of LTPWM-III. LTPWM-III was not blocked in the contralateral, noninfused visual cortex. These data suggest that one function of the REMS state in early maturation may be to facilitate the development of cortical inhibitory processes involved with the closure of the CP, possibly by upregulating expression of the neurotrophin BDNF.

To this point, these data support Howard’s ontogenetic hypothesis that REMS state activation during development, like visual stimulation, has promotes maturation, and like removal of visual stimulation, REMSD effectively delays maturation in the visual system. Howard’s group then decided to determine whether REMS also influences maturation of a brain structure that is not *directly* in the path of exogenous sensory input, the hippocampus.

To explore whether REMS affects other brain areas besides the visual system, they turned to a comprehensive study by Kramar and Lynch [57] showing that hippocampal LTP stability is developmentally regulated; once LTP is produced in vitro, it is susceptible to disruption in very young animals (PN 9-21) but in older animals, LTP, once produced, is resilient to countervalent challenges. LTP instability also was shown to be associated with immature and unstable synapses [57]. The Kramar and Lynch model was adopted to investigate the effect of REMSD on the maturation of the hippocampus. These studies showed that in young rats 4 h of REMSD on three consecutive days (P16–18, 12 h total) did not interfere with LTP induction in hippocampus at P21–25, but the stability of hippocampal LTP was significantly reduced in these animals [57]. LTP stability was reduced in the P21–25 REMSD group *compared to* all other groups, including the P49–53 REMSD, the P21–25, and P49–53 normal groups. LTP instability in the P21–25 REMSD group brought LTP back to nearly baseline levels after being challenged, much as in the untreated, immature (P9–12) group. These in vitro LTP studies were conducted for at least 3 days and as long as 7 days after the third 4-hour REMSD period in the P21–25 rats, with recovery sleep after each REMSD session. It is significant that the tissue retained the REMSD effect postmortem.

Since synaptic composition of glutamatergic synapses is regulated by activity during development [58, 59], tissue from the contralateral hippocampus in each of the same animals was assessed for receptors and associated proteins. Immunoblots assayed protein levels for several glutamatergic receptor subunits (*N*-methyl-D-aspartate receptor subunit 2, NR2A and NR2B); an AMPA receptor subunit (GluR1) that is strongly regulated by the intensity of synaptic activity [60]; and an associated synaptic membrane molecule (PSD-95, postsynaptic density protein 95), which is a scaffolding protein of the postsynaptic density matrix involved in anchoring and linking NMDARs to intracellular signaling pathways [61]. Because in vivo analyses have demonstrated a large increase in calcium/calmodulin kinase II (CaMKII) expression during P10–35, and this expression of CaMKII correlates with spine production and spine stability [62], protein levels of this molecule were also measured.

Immunoblot analyses showed changes in several glutamatergic receptor subunits and downstream signaling molecules. Overall, in the PN21-25 REMSD animals, NR2B, NR2A, GluR1, PSD-95, and CaMKII levels were, without exception, lower (NR2B, GluR1, and PSD-95 were significantly lower) compared to age-matched controls. Effects of REMSD on these glutamatergic signaling proteins were not observed in the P46–58 REMSD animals. The immunoblot data, therefore suggested that the effects of early-life REMSD on stability of hippocampal neuronal circuits are linked to reduced expression of glutamatergic synaptic components [63].

These data connect suppression of REMS to the changes in the molecular constituents of synaptic plasticity in hippocampus. By implication, this consequence of REMSD prompts a consideration that these signaling molecules are key mechanisms contributing to how the REMS state affects brain maturation [63]. The results also demonstrate that during development REMS contributes activation to brain areas that do not directly receive external sensory stimulation as well as to sites primarily engaged in sensory processing. The findings are in general agreement with the kitten and rat studies that showed delayed maturation in visual cortex. Overall, the results lend strong support for Howard’s ontogenetic hypothesis by implicating the activity of the REMS state having a role in the mechanisms that contribute to brain maturation [19, 20, 26, 32, 64].

Howard was the originator of what has come to be known as the “Ontogenetic Hypothesis.” His physiologically based research over many years has contributed greatly to much of the experimental support substantiating a role for REMS in early development of the brain. Though much is still unknown, the ontogenetic hypothesis has, and is still to this day, inspired many neuroscientists in their investigations. These studies have demonstrated roles for both REMS and NREMS in development as well as influences on brain function throughout the life span.

Last, but certainly not least, is Howard’s contribution to sleep disorders medicine. Early in his career, Howard came somewhat late to the study of sleep disorders medicine. However, as more and more of his colleagues in the sleep research community embraced this new branch of medicine, his enthusiasm grew rapidly. Howard served as chairman of Sleep Disorders Classification Committee, American Sleep Disorders Association, which led to the creation of the first, consensus, sleep-medicine nosological system, “The Diagnostic Classification of Sleep and Arousal Disorders” (1979). In the 1980s, he chaired the American Psychiatric Association DSM-III-R Committee on Sleep Disorders Diagnosis. He was vice-President of the American Sleep Disorders Association and in the 1990s, Director of the American Board of Sleep Medicine. In the 2000s, he was a member of the NIH Sleep Disorders Research Advisory Board. Howard also administrated the practice of sleep disorders medicine and directed the sleep disorders clinics at UT Southwestern (this was the first sleep clinic founded in the north Texas region), and the UMMC School of Medicine. Howard apparently made up for starting a little late.

## Concluding Remarks

In Howard’s conference room in Dallas, there was a framed placard of a Chinese fortune cookie he had received at dinner at the Red Maple Chinese Restaurant in NYC in 1977. It read “You could prosper in the field of biomedical research.” Truly, it can be said, Howard P. Roffwarg had a prosperous career in biomedical research. It spanned his multiple roles as a student and mentor, clinician, leadership role in the SRS, work in sleep medicine, experimental investigations in humans and animals, and his enduring pursuit of testing the “Ontogenetic Hypothesis.” Dr Howard P. Roffwarg, MD, is one of the legends the field of sleep research.

Howie passed away in October 2022, just prior to the publishing of this article. We like to believe, with apologies to Raymond Chandler, that Howie is now studying the Big Sleep. May his soul rest in peace and discover all the mysteries of Sleep.

Howie Roffwarg, our beloved mentor, colleague and friend,

Jim and Gerry

## Data Availability

All the data referenced herein have been previously published and referenced in the references section.

## Notes

This paper is part of the Living Legends in Sleep Research series, which is sponsored by Idorsia Pharmaceuticals and Jazz Pharmaceuticals.

## Editor’s Note

Before his death, Dr Roffwarg agreed to be featured in the journal’s Living Legend series, with the assistance of Drs Shaffery and Marks write of his career in sleep.

## References

[1] Aserinsky E , et al. Regularly occurring periods of eye motility and concomitant phenomena during sleep. Science. 1953;118:273–274. doi:10.1126/science.118.3062.273 13089671

[2] Pace-Schott EF , et al. The neurobiology of sleep: genetics, cellular physiology and subcortical networks. Nat Rev Neurosci. 2002;3(8):591–605. doi:10.1038/nrn895 12154361

[3] Steriade M , et al. Neuronal activity during the sleep-waking cycle. Prog Neurobiol. 1976;6(3–4):155–376. 6996

[4] Jouvet-Mounier D , et al. Ontogenesis of the states of sleep in rat, cat, and guinea pig during the first postnatal month. Dev Psychobiol. 1970;2(4):216–239. 5527153 10.1002/dev.420020407

[5] Siegel JM. Sleep function: an evolutionary perspective. Lancet Neurol. 2022;21(10):937–946. doi:10.1016/s1474-4422(22)00210-1 36115365 PMC9670796

[6] Roffwarg HP , et al. Ontogenetic development of the human sleep-dream cycle. Science. 1966;152:604–619. doi:10.1126/science.152.3722.604 17779492

[7] Shaffery JP. Sleep and brain development. In: Dringenberg HC, ed. Handbook of Sleep Research. Vol 30. Amsterdam: Academic Press; 2019: 413–424.

[8] Rechtschaffen A , Kales A. A manual of Standardized Terminology,Techniques and Scoring System for Sleep Stages of Human Subjects. Public Health Service, U.S. Printing Office; 1968.

[9] Farber J , et al. Rapid eye movement sleep PGO-type waves are present in the dorsal pons of the albino rat. Science. 1980;209(4456):615–617. doi:10.1126/science.6994229 6994229

[10] Marks GA , et al. Demonstration of ponto-geniculo-occipital waves in the albino rat. Exp Neurol. 1980;69(3):648–666. 6447612 10.1016/0014-4886(80)90058-8

[11] Weaver KJ , et al. Neonatal exposure to citalopram selectively alters the expression of the serotonin transporter in the hippocampus: dose-dependent effects. Anat Rec. 2010;293(11):1920–1932. 10.1002/ar.21245 PMC2967660 20830689

[12] Maciag D , et al. Neonatal antidepressant exposure has lasting effects on behavior and serotonin circuitry. J Neuropsychopharmacol. 2006;31(1):47–57. 10.1038/sj.npp.1300823 PMC3118509 16012532

[13] Hubel DH , et al. Single-cell responses in striate cortex of kittens deprived of vision in one eye. J Neurophysiol. 1963;26:1003–1017. 14084161 10.1152/jn.1963.26.6.1003

[14] Wiesel TN , et al. Effects of visual deprivation on morphology and physiology of cells in the cat’s lateral geniculate body. J Neurophysiol. 1963;26:978–993. 14084170 10.1152/jn.1963.26.6.978

[15] Mower GD , et al. Role of visual experience in activating critical period in cat visual cortex. J Neurophysiol. 1985;53(2):572–589. doi:10.1152/jn.1985.53.2.572 3981230

[16] Mendelson WB , et al. The flower pot technique of rapid eye movement (REM) sleep deprivation. Pharmacol Biochem Behav. 1974;2:553–556. 4371007 10.1016/0091-3057(74)90018-5

[17] Oksenberg A , et al. Effect of REM sleep deprivation during the critical period. Sleep Res. 1986;15:53.

[18] Oksenberg A , et al. Testing the role of rapid eye movement (REM) sleep in the development of the CNS. Sleep Res. 1985;14:76.

[19] Oksenberg A , et al. Rapid eye movement sleep deprivation in kittens amplifies LGN cell-size disparity induced by monocular deprivation. Brain Res Dev Brain Res. 1996;97(1):51–61. 8946054 10.1016/s0165-3806(96)00131-9

[20] Shaffery JP , et al. REM sleep deprivation in monocularly occluded kittens reduces the size of cells in LGN monocular segment. Sleep. 1998;21(8):837–845. doi:10.1093/sleep/21.8.837 9871946

[21] Datta S. Cellular basis of pontine ponto-geniculo-occipital wave generation and modulation. Cell Mol Neurobiol. 1997;17(3):341–365. 9187490 10.1023/A:1026398402985 PMC11560215

[22] Steriade M , et al. Neuronal activities in brain-stem cholinergic nuclei related to tonic activation processes in thalamocortical systems. J Neurosci. 1990;10(8):2541–2559. 2388079 10.1523/JNEUROSCI.10-08-02541.1990 PMC6570275

[23] Davenne D , et al. Suppression of PGO waves in the kitten: anatomical effects on the lateral geniculate nucleus. Neurosci Lett. 1984;45:33–38. 6728304 10.1016/0304-3940(84)90325-2

[24] Davenne D , et al. Lesion of the PGO pathways in the kitten. II. Impairment of physiological and morphological maturation of the lateral geniculate nucleus. Brain Res. 1989;485(2):267–277. doi:10.1016/0006-8993(89)90570-2 2720412

[25] Davenne D , et al. Lesion of the ponto-geniculo-occipital pathways in kittens. I. Effects on sleep and unitary discharge of the lateral geniculate nucleus. Brain Res. 1987;409:1–9. doi:10.1016/0006-8993(87)90735-9 3580860

[26] Shaffery JP , et al. Ponto-geniculo-occipital-wave suppression amplifies lateral geniculate nucleus cell-size changes in monocularly deprived kittens. Brain Res Dev Brain Res. 1999;114(1):109–119. 10209248 10.1016/s0165-3806(99)00027-9

[27] Friedlander MJ , et al. Effects of monocular visual deprivation on geniculocortical innervation of area 18 in cat. J Neurosci. 1991;11(10):3268–3288. doi:10.1523/jneurosci.11-10-03268.1991 1941084 PMC6575440

[28] Demeulemeester H , et al. Calcium binding proteins as molecular markers for cat geniculate neurons. Exp Brain Res. 1991;83(3):513–520. 2026194 10.1007/BF00229828

[29] Demeulemeester H , et al. Calcium binding proteins and neuropeptides as molecular markers of GABAergic interneurons in the cat visual cortex. Exp Brain Res. 1991;84(3):538–544. 1864325 10.1007/BF00230966

[30] Hogan D , et al. The effects of 1 week of REM sleep deprivation on parvalbumin and calbindin immunoreactive neurons in central visual pathways of kittens. J Sleep Res. 2001;10(4):285–296. 11903858 10.1046/j.1365-2869.2001.00270.x

[31] Caillard O , et al. Role of the calcium-binding protein parvalbumin in short-term synaptic plasticity. Proc Natl Acad Sci USA. 2000;97(24):13372–13377. doi:10.1073/pnas.230362997 11069288 PMC27231

[32] Shaffery JP , et al. Rapid eye movement sleep deprivation modifies expression of long-term potentiation in visual cortex of immature rats. Neuroscience. 2002;110(3):431–443. 11906784 10.1016/s0306-4522(01)00589-9

[33] Kirkwood A , et al. Co-regulation of long-term potentiation and experience-dependent synaptic plasticity in visual cortex by age and experience. Nature. 1995;375(6529):328–331. doi:10.1038/375328a0 7753198

[34] Zhou Q , et al. Reversal and stabilization of synaptic modifications in a developing visual system. Science. 2003;300(5627):1953–1957. doi:10.1126/science.1082212 12817152

[35] Rittenhouse CD , et al. Monocular deprivation induces homosynaptic long-term depression in visual cortex. Nature. 1999;397(6717):347–350. doi:10.1038/16922 9950426

[36] Rozas C , et al. Developmental inhibitory gate controls the relay of activity to the superficial layers of the visual cortex. J Neurosci. 2001;21(17):6791–6801. doi:10.1523/jneurosci.21-17-06791.2001 11517267 PMC6763109

[37] Kirkwood A , et al. Elementary forms of synaptic plasticity in the visual cortex. Biol Res. 1995;28(1):73–80. 8728822

[38] Kirkwood A , et al. Homosynaptic long-term depression in the visual cortex. J Neurosci. 1994;14(5 Pt 2):3404–3412. 8182481 10.1523/JNEUROSCI.14-05-03404.1994 PMC6577491

[39] Kirkwood A , et al. Common forms of synaptic plasticity in the hippocampus and neocortex in vitro. Science. 1993;260:1518–1521. doi:10.1126/science.8502997 8502997

[40] Fagiolini M , et al. Inhibitory threshold for critical-period activation in primary visual cortex. Nature. 2000;404(6774):183–186. doi:10.1038/35004582 10724170

[41] Hensch TK , et al. Comparison of plasticity in vivo and in vitro in the developing visual cortex of normal and protein kinase A RIbeta-deficient mice. J Neurosci. 1998;18(6):2108–2117. 9482797 10.1523/JNEUROSCI.18-06-02108.1998 PMC2553093

[42] Kirkwood A , et al. Experience-dependent modification of synaptic plasticity in visual cortex. Nature. 1996;381(6582):526–528. 8632826 10.1038/381526a0

[43] Kato N , et al. Developmental changes in the susceptibility to long-term potentiation of neurons in rat visual cortex slices. Brain Res Dev Brain Res. 1991;60(1):43–50. 1680581 10.1016/0165-3806(91)90153-a

[44] Perkins AT , et al. A critical period for long-term potentiation in the developing rat visual cortex. Brain Res. 1988;439(1–2):222–229. 3359185 10.1016/0006-8993(88)91478-3

[45] Sermasi E , et al. A new form of synaptic plasticity is transiently expressed in the developing rat visual cortex: a modulatory role for visual experience and brain-derived neurotrophic factor. Neuroscience. 1999;91(1):163–173. doi:10.1016/s0306-4522(98)00598-3 10336067

[46] Maffei L , et al. Nerve growth factor (NGF) prevents the shift in ocular dominance distribution of visual cortical neurons in monocularly deprived rats. J Neurosci. 1992;12(12):4651–4662. 1334503 10.1523/JNEUROSCI.12-12-04651.1992 PMC6575769

[47] Artola A , et al. Long-term potentiation and NMDA receptors in rat visual cortex. Nature. 1987;330(6149):649–652. doi:10.1038/330649a0 2446147

[48] Pesavento E , et al. Blocking the NGF-TrkA interaction rescues the developmental loss of LTP in the rat visual cortex: role of the cholinergic system. Neuron. 2000;25(1):165–175. doi:10.1016/s0896-6273(00)80880-6 10707981

[49] Mower GD , et al. The effects of dark-rearing on the development and plasticity of the lateral geniculate nucleus. Brain Res. 1981;227(3):418–424. 7260648 10.1016/0165-3806(81)90079-1

[50] Mower GD , et al. Comparison of the effects of dark rearing and binocular suture on development and plasticity of cat visual cortex. Brain Res. 1981;220(2):255–267. doi:10.1016/0006-8993(81)91216-6 7284755

[51] Mower GD , et al. Dark rearing prolongs physiological but not anatomical plasticity of the cat visual cortex. J Comp Neurol. 1985;235(4):448–466. doi:10.1002/cne.902350404 3998219

[52] Morden B , et al. Selective REM sleep deprivation and compensation phenomena in the rat. Brain Res. 1967;5(3):339–349. doi:10.1016/0006-8993(67)90042-x 6035939

[53] Shaffery JP , et al. Rapid eye movement sleep deprivation revives a form of developmentally regulated synaptic plasticity in the visual cortex of post-critical period rats. Neurosci Lett. 2006;391(3):96–101. doi:10.1016/j.neulet.2005.08.044 16154270

[54] Aizenman CD , et al. A current source density analysis of evoked responses in slices of adult rat visual cortex: implications for the regulation of long-term potentiation. Cereb Cortex. 1996;6(6):751–758. doi:10.1093/cercor/6.6.751 8922331

[55] Huang ZJ , et al. BDNF regulates the maturation of inhibition and the critical period of plasticity in mouse visual cortex. Cell. 1999;98(6):739–755. doi:10.1016/s0092-8674(00)81509-3 10499792

[56] Inagaki T , et al. Brain-derived neurotrophic factor-mediated retrograde signaling required for the induction of long-term potentiation at inhibitory synapses of visual cortical pyramidal neurons. Neurosci Res. 2008;61(2):192–200. doi:10.1016/j.neures.2008.02.006 18395922

[57] Kramar EA , et al. Developmental and regional differences in the consolidation of long-term potentiation. Neuroscience. 2003;118(2):387–398. 12699775 10.1016/s0306-4522(02)00916-8

[58] Desai NS , et al. Critical periods for experience-dependent synaptic scaling in visual cortex. Nat Neurosci. 2002;5(8):783–789. 12080341 10.1038/nn878

[59] Perez-Otano I , et al. Learning from NMDA receptor trafficking: clues to the development and maturation of glutamatergic synapses. Neurosignals. 2004;13(4):175–189. doi:10.1159/000077524 15148446

[60] Passafaro M , et al. Induction of dendritic spines by an extracellular domain of AMPA receptor subunit GluR2. Nature. 2003;424(6949):677–681. doi:10.1038/nature01781 12904794

[61] Sans N , et al. A developmental change in NMDA receptor-associated proteins at hippocampal synapses. J Neurosci. 2000;20(3):1260–1271. doi:10.1523/jneurosci.20-03-01260.2000 10648730 PMC6774158

[62] Petralia RS , et al. Ontogeny of postsynaptic density proteins at glutamatergic synapses. Mol Cell Neurosci. 2005;29(3):436–452. 15894489 10.1016/j.mcn.2005.03.013 PMC1414063

[63] Lopez J , et al. Rapid eye movement sleep deprivation decreases long-term potentiation stability and affects some glutamatergic signaling proteins during hippocampal development. Neuroscience. 2008;153(1):44–53. doi:10.1016/j.neuroscience.2008.01.072 18359575 PMC2389877

[64] Marks GA , et al. A functional role for REM sleep in brain maturation. Behav Brain Res. 1995;69(1–2):1–11. 10.1016/0166-4328(95)00018-o 7546299

